# Remote Sensing of Grassland Plant Biodiversity and Functional Traits

**DOI:** 10.1002/ece3.71829

**Published:** 2025-07-28

**Authors:** Samuel Hayes, Fiona Cawkwell, Karen L. Bacon, Astrid Wingler

**Affiliations:** ^1^ School of Biological, Earth and Environmental Sciences University College Cork Cork Ireland; ^2^ Sustainability Institute University College Cork Cork Ireland; ^3^ Department of Geography University College Cork Cork Ireland; ^4^ Botany and Plant Science University of Galway Galway Ireland

**Keywords:** functional traits, grasslands, hyperspectral, multispectral, species richness, spectral variation hypothesis

## Abstract

The use of remotely sensed imagery for the monitoring of both plant biodiversity and functional traits in grassland ecosystems has increased substantially in the last few decades. More recently, uncrewed aerial vehicles (UAVs) have begun to play an increasingly important role, providing repeatable very high‐resolution data, acting as a bridge between the decameter satellite imagery and the point scale data collected on the ground. At the same time, machine learning approaches are rapidly expanding, adding new analysis and modeling tools to the plethora of UAV, aircraft, and satellite observational data. Here, we provide a review of remotely sensed monitoring methods for grassland plant biodiversity and functional traits (Leaf Dry Matter Content, Crude Protein, Potassium, Phosphorus, Nitrogen and Leaf Area Index) between 2018 and 2024. We highlight the key innovations that have occurred, sources of error identified, new analysis methods presented, and identify the bottlenecks to and opportunities for further development. We emphasize the need for (1) the integration of observations across spatial and temporal scales, (2) a more systematic identification and examination of sources of error and uncertainty, (3) more widespread use of hyperspectral satellite data, and (4) greater focus on the development of a grassland global spectra database—linking spectra, species diversity metrics, and functional traits.

## Graphical abstract

Here we review developments in the remote sensing of grassland plant biodiversity and functional traits from 2018 to 2024. New tools, technology, and methods are presented, their uncertainties, limitations, and potential highlighted, and recommendations are provided for future directions to help make the most of this rapidly changing and growing area of research.

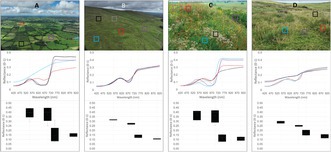

## 1 Introduction

Grasslands cover 30% to 40% of the Earth's land surface (Blair et al. 2014) and are responsible for up to a third of net primary productivity on land (Vitousek 2015), providing many important ecosystem services, from water flow regulation and purification to erosion control and pollination (Bengtsson et al. 2019; Peciña et al. 2019). Grasslands also contribute significantly to livestock farming through grazing and fodder production (Erb et al. 2016).

Natural and semi‐natural grasslands are often characterised by high community complexity (Wilson et al. 2012), making them important sources of, and contributors to, plant biodiversity (Russo et al. 2022). Surveys carried out on experimental plots have shown that increased grassland biodiversity can contribute to greater yields, improved yield stability, and increased carbon sequestration (Craven et al. 2018; Finn et al. 2013; Haughey et al. 2018; Isbell et al. 2015; Lange et al. 2015). However, through land‐use change, abandonment, urbanisation, and intensive agriculture, natural and semi‐natural grasslands have become endangered ecosystems (Johansen et al. 2022; Pärtel et al. 2005) with decreases in their area and reductions in their biodiversity in recent decades (Henle et al. 2008; O'Mara 2012; Newbold et al. 2016).

Plant functional traits (biochemical, physical and morphological properties that affect fitness in response to the environment) and trait diversity are key features of (semi‐)natural grasslands. For example, traits such as high leaf dry matter content (LDMC), low specific leaf area, and low leaf nitrogen content indicate stress tolerance strategies of grass species and adaptation to low temperature and low precipitation (Wingler and Sandel 2023). The relationship between such plant functional traits and their role in ecosystem functioning and ecosystem services (e.g., water regulation, carbon storage, stress tolerance) is well‐established (Kattge et al. 2011; Tilman et al. 1997).

**FIGURE 1:**
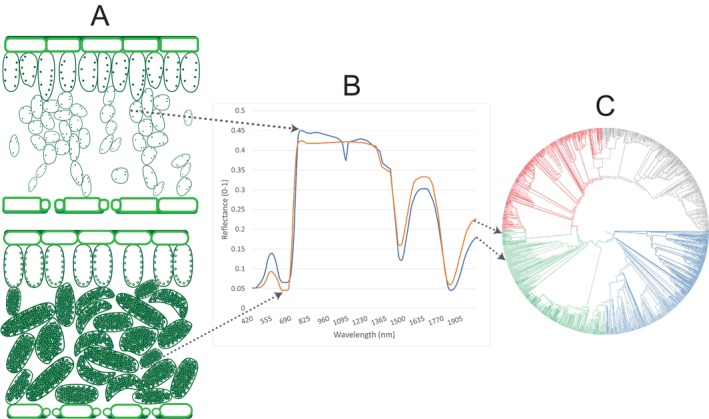
How the (A) physical and chemical properties of plants influence their interaction with light, producing (B) a recognisable spectral signature that can aid in determining the plant species (C) Adapted from Cavender‐Bares et al. (2017).

Optical remote sensing offers the ability to monitor biodiversity and functional traits across a range of scales, from centimetres to kilometres, in a consistent and repeatable manner. The physical and chemical properties of plants influence how sunlight interacts with them. By examining the absorption and reflection of light across different parts of the electromagnetic spectrum, information about the species diversity (Figure 1) (Wang and Gamon 2019), functional traits (Homolová et al. 2013) and thus α‐diversity (diversity at a local scale) and β‐diversity (ratio between regional and local diversity) can be extracted.

Recent advances in the spatial, spectral, and temporal resolution of satellite sensors make them increasingly suited to grassland monitoring, even in the relatively small and fragmented grasslands that occur in regions such as Europe. Additionally, instruments can be mounted on aircraft to provide multispectral (typically up to a dozen discrete spectral bands) or hyperspectral (100 s or > 1000 discrete spectral bands) data at a spatial resolution of under a meter. Furthermore, developments in Uncrewed Aerial Vehicle (UAV) technology now allow similar data to be captured at spatial resolutions down to millimeters.

The tools and monitoring techniques across multiple spatial and spectral scales have developed rapidly in recent years, requiring timely reviews of the current state of research. Such reviews ensure that land managers and researchers are kept appraised of the tools and techniques available to preserve current (semi‐)natural grasslands, protect biodiversity, and ensure the continuation of important ecosystem services. To this end, we aim to provide an overview of the recent progress in the remote sensing of grassland plant biodiversity and six above‐ground functional traits. While biodiversity is complex and can be represented and analyzed through a broad range of measures, here we primarily focus on species richness and species diversity metrics when discussing grassland plant biodiversity, with the specific metric employed within a study described as needed.

The six functional traits were chosen to reflect different aspects of grassland functional diversity and different prevalences within the research literature. This allows for an assessment of progress in both established trait measurements and advancements in less commonly measured traits: (1) The Leaf Area Index (LAI) a very commonly measured vegetation structural trait that is important for productivity and forms the basis for many other functional trait observations. (2) Leaf Dry Matter Content (LDMC) is an important factor in productivity, forage quality, and resilience, but is much under‐studied in comparison to LAI. (3) Nitrogen (N) is vital for plant growth and productivity and is very commonly measured. (4) Phosphorus (P) and Potassium (K) are vital micronutrients for metabolic activity that have been measured in relatively few previous remote sensing studies. (5) Crude protein (CP) is an important aspect of productivity and forage quality in grasslands and is also commonly measured within the remote sensing literature.

## 2 Literature Search and Filtering Criteria

**FIGURE 2:**
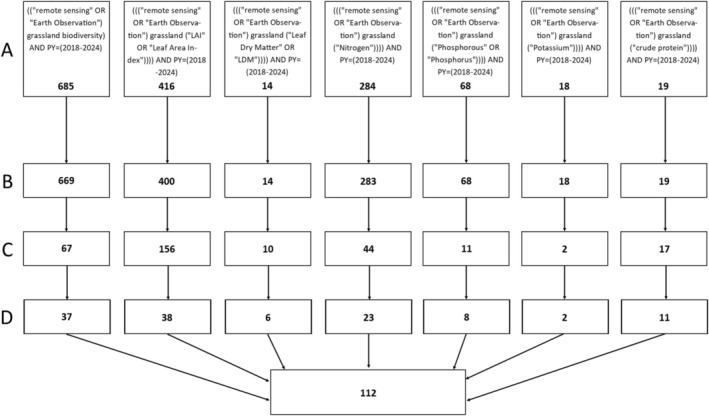
(A) the search terms used and number of papers, (B) filter by research and reviews, (C) examination of abstracts for keywords, and (D) checking of the papers to ensure they represent a novel method, development of a method, or accuracy analysis of a remote sensing method.

For this review, a literature search was performed on the core collection of the Web of Science database for the Years 2018–2024. This date range keeps focus on the recent and relevant advances in what is a rapidly developing field. One search was conducted for biodiversity, and one for each of the six selected functional traits. The search terms used in Figure 2A produced the first round of results, ranging from 685 for biodiversity and 14 for leaf dry matter, and a total of 1504 results. The first filter, row B in Figure 2, selected just research articles and reviews, which reduced the total by 2.2%. The second filter, row C in Figure 2, examined the abstracts to ensure that the papers were directly related to the search theme. This removed 77.4% of the original total. The final filter, row D in Figure 2, involved examination of the papers to ensure they represented a development or advancement in the remote sensing methods, or an analysis/assessment of the remote sensing methods. Furthermore, papers that exclusively used proximal sensors, such as handheld spectrometers, rather than remote sensors, were also excluded. This brought the total number of papers down to 125, with a final total of 112 after duplicates were removed.

## 3 Remote Sensing of Grassland Biodiversity

**FIGURE 3:**
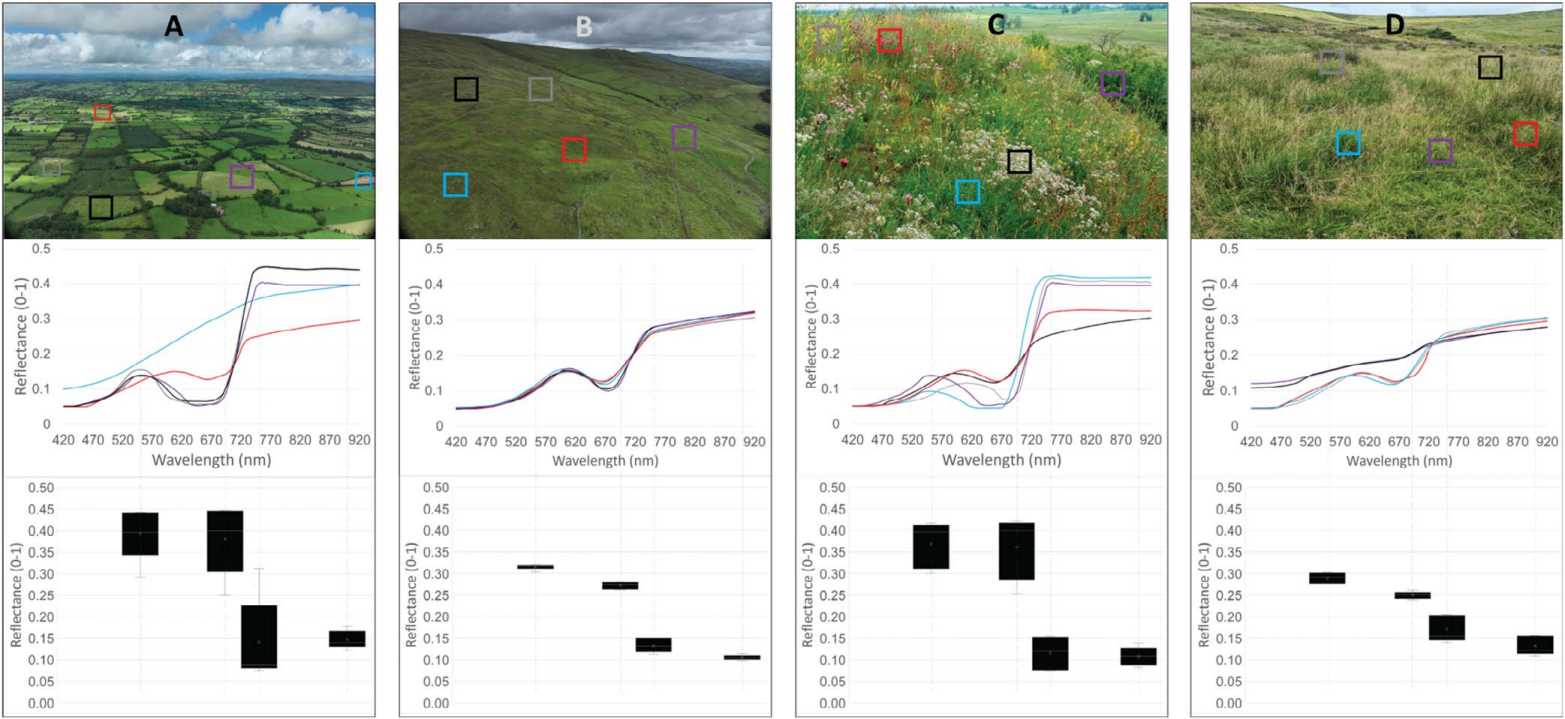
The top panel shows aerial views depicting beta diversity in a diverse landscape (A) and a low‐diversity landscape (B), and alpha diversity in and a highly diverse grassland (C) and a low‐diversity grassland (D). The second panel shows the spectral signatures associated with the coloured boxes on the top panel. The bottom panel shows the corresponding spectral diversity in the green, red, red‐edge and near‐infrared wavelengths, from left to right, respectively.

This section will be split into three broad categories. The first is based on the spectral variation hypothesis (SVH). This is the most common method for mapping plant biodiversity and is centered on the premise that individual plant species absorb and reflect sunlight in unique ways, creating a distinct spectral signature (Figure 3). Where there are many distinct species in a grassland, the spectral diversity (SD) recorded by the remote sensing instrument will be greater than in areas with fewer species (Rocchini et al. 2004). This type of analysis can be performed with both multi‐ and hyper‐spectral instruments, with measures of SD ranging from simple standard deviations of spectral bands to convex hull volumes of the principal components of hundreds of hyperspectral bands and more. Studies utilizing the SVH approach are the focus of 18 of the 37 biodiversity papers in this section, representing refinement of the methodology, application in different environments, as well as exploration of mediating factors and limitations.

The second biodiversity category encompasses studies with a focus on machine learning. As in many scientific fields, remote sensing of grassland biodiversity has experienced an accelerated uptake in the use of machine learning in the last few years. Here, they account for 12 of the 38 papers presented.

The third category explores studies that focus on neither the SVH nor machine learning (although they form small parts of some studies) but include approaches from manual identification of species from UAV imagery to interdisciplinary research.

### 3.1 Spectral Variation Hypothesis

The SVH has been employed across a broad range of spatial scales, yet there appears to be little consistency regarding the ideal spatial resolution at which it best operates. At an experimental prairie site, Wang et al. (2018) found the ideal pixel size to be between 1 mm and 10 cm to establish a strong relationship between species richness and spectral diversity, with the relationship fading after 10 cm. Similarly, Polley et al. (2019) suggest that the sensitivity of SD to species diversity is scale dependent and that the necessary spectral details may be lost with greater spatial scales. Gholizadeh et al. (2019) found a strong relationship between SD and species richness at both 0.5 m and 1 m resolution, but not at 5 m. However, Gholizadeh, Friedman, et al. (2022) failed to connect SD to species richness at both 1 m and 30 m, only achieving significant correlations with the Simpsons diversity index. The authors also highlight that ground sampling design also impacts the relationship between biodiversity and SD. Similarly, no strong correlations were found between species richness and a large range of standard SD metrics at 2 cm and 5 cm pixel sizes from multispectral UAV surveys (Perrone et al. 2024). The authors suggest that phenological stage plays a key role, and finer resolutions can be more noisy due to factors like shadowing. Jackson et al. (2022) used a multispectral UAV to estimate biodiversity at 0.1–0.5 cm resolution. They could predict the Shannon–Weiner and Simpson's biodiversity indices well, but not species richness. They note that the measure of SD decreased by 30% with every additional 1 m in elevation that the UAV was flown.

Confounding factors that influence how well SD is related to biodiversity have been explored on a variety of grasslands in recent years. Yang, Lei, et al. (2023) noted how an open‐pit coal mine influenced α‐, β‐, and γ‐ diversity in an Inner Mongolian steppe, and that grazing increased the area that was most strongly affected. Using Landsat data for Himalayan grasslands, Chitale et al. (2019) explained that variance in species richness measured using vegetation indices increased from 54% to 85% after the inclusion of physiographic indices. In more general terms, it has been found that accounting for the effects of bare soil on the spectral readings can significantly improve remote sensing estimations of both α‐ and β‐ diversity (Kamaraj et al. 2024; Xu et al. 2022). In contrast to many other studies, Conti et al. (2021) found a negative relationship between SD and taxonomic diversity in mesic meadows in Czechia, with the vertical complexity driving the relationship—the more vertically complex the grass structure, the more negative the relationships between SD and taxonomic diversity. In addition, the timing of flowering plants (Perrone et al. 2024), the presence of non‐native species (Van Cleemput et al. 2023), recent fires (Gholizadeh et al. 2020) and the proportion of live and dead biomass (Rossi et al. 2022) have all now been identified as confounding factors.

Several studies have explored the use of time series analysis to overcome uncertainties in the SD/biodiversity relationship. Rossi et al. (2021) introduced a spatio‐temporal version of Rao's quadratic entropy index (RaoQ) to examine changes in β‐diversity over time with Sentinel‐2 imagery and account for variations in grassland management and phenology. They suggest that, with higher resolution data, this method can be applied to α‐diversity too. In exploring how α‐ and β‐diversity vary over 2 years in prairie grassland, Gholizadeh et al. (2020) found significant differences in species richness, due to factors such as fires and weather, and recommend multi‐temporal surveys to account for these changes. When assessing grasslands in the USA and Europe, Rossi et al. (2024), with a Sentinel‐2 timeseries, found a stronger and more consistent relationship with species diversity from temporal SD than spatial SD, suggesting that an analysis based on a single snapshot in time can be misleading.

Some new approaches have also attempted to tackle these uncertainties. Zhao, Sun, Chen, et al. (2021) used cluster analysis of hyperspectral data to identify distinct spectral species, allowing them to accurately predict plant species diversity (*R* ^2^ of 0.73). Developing this idea further, Rossi and Gholizadeh (2023) used spectral unmixing. They first determined the number of distinct spectral entities, called endmembers, within each image. Then they calculated the number of endmembers and their abundance within each pixel and used that information to create endmember spectral diversity metrics. The authors claim that this approach is less sensitive to soil and can also be applied to multi‐temporal datasets, which may help to overcome some of the previously identified confounding factors.

### 3.2 Machine Learning

To assess plant species diversity over part of the Tibetan Plateau, Zhao et al. (2022) used high‐accuracy surface modelling (HASM), Landsat‐8 data, and a range of machine learning models, namely least absolute shrinkage and selection operator, ridge regression, eXtreme Gradient Boosting, and Random Forest (RF). The authors found that the models combined with HASM performed better than the machine learning models alone, with the best combination being eXtreme Gradient Boosting and HASM, followed closely by RF and HASM. Fauvel et al. (2020) experimented with combining the multispectral data of Sentinel‐2 with the radar from Sentinel‐1 to map biodiversity in grasslands in southern France with multiple regression methods—Linear regression, K‐Nearest Neighbours, Kernel Ridge Regression, RF, and Gaussian Process. They found that RF worked best overall, with *R* ^2^ values above 0.4 for the Simpson and Shannon indices, and the addition of Sentinel‐1 data provided no significant improvements. Another attempt to combine Sentinel‐1 and ‐2 came from Muro et al. (2022) with RF and deep neural networks employed to predict biodiversity. The deep neural networks model performed slightly better than RF, though both performed poorly under cross‐validation, and the addition of Sentinel‐1 again provided little benefit. Several other studies found success with mapping plant species diversity using different forms of neural networks. In semi‐natural meadows in Germany, convoluted neural networks were used to classify multispectral UAV data, mapping vegetation units with accuracies of up to 88% (Pöttker et al. 2023). In three distinct German grasslands, a residual neural network model was used with a time series of Sentinel‐2 data to map a range of plant biodiversity metrics, achieving *R* ^2^ values of up to 0.68 and showing significant improvements in accuracy compared to other machine learning methods assessed (Dieste et al. 2024). Employing convoluted neural networks in a different way, Gallmann et al. (2022) managed to identify and count individual flowers from images taken by drone‐mounted standard high‐resolution camera, performing as well or better than manual counting for most flower species.

However, RF tended to produce the most accurate results for the majority of studies. Using weather data and MODIS‐based Normalised Difference Vegetation Index (NDVI) over Tibet, Tian and Fu (2022) found RF to produce the most accurate measures of plant species diversity compared to numerous other machine learning methods. Again, over the Tibetan Plateau and using MODIS data (and weather, soil and topographic variables) Yang, Chen, et al. (2023) achieved an *R* ^2^ of 0.6 for plant species diversity with RF after using stepwise regression for variable selection. In mountainous grasslands in South Africa, Mashiane et al. (2024) used vegetation indices (VIs) from Sentinel‐2 and Landsat‐8 and RFs to model species richness and the Shannon–Wiener index, achieving *r* ^2^ values above 0.85 for both. RF modeling was also most accurate compared to other machine learning methods in the Three Rivers Headwater Region of China, where Yang et al. (2024) used stepwise regression to select among variables from Landsat, climate, soil, and topographic data. Indeed, using VIs, canopy height, and textural data derived from multispectral UAV surveys over two summers in a wet grassland near Berlin, Bazzo et al. (2024) also found RF modeling to produce the most accurate and consistent measures of species richness. Finally, in an assessment of plant diversity in numerous ecosystems across the world (including 315 grassland plots), Xin et al. (2024) again found that RF models produced the most consistent and accurate results compared to other regression and machine learning models.

### 3.3 Other Methods

Some researchers have taken slightly different approaches to remotely mapping grassland biodiversity. Löfgren et al. (2018) attempted to use both satellite and UAV‐based NDVI values to map the richness of specialist species in grasslands on a Baltic island in southern Sweden, but achieved only weak, negative correlations. In the alpine grasslands of Tibert, Qin et al. (2020) achieved more significant correlations between richness, Shannon, Simpson, and Pielou's indices derived from the manual counting of species from UAV imagery versus traditional quadrat surveys, and identified more species (71) from UAV imagery than from quadrat surveys (63). Another study on the Tibetan Plateau found a significant relationship between UAV‐measured bare patches in grasslands with decreases in richness and increased species turnover (Hua et al. 2023). In mountainous grasslands of northern Portugal, Monteiro et al. (2021) found that the NIR/Green ratio values from Sentinel‐2 and their seasonal amplitude correlated well with species richness, producing an *R* ^2^ of 0.44. In tallgrass prairies in the USA, Hall and Lara (2022) compared combinations of hyperspectral UAV and LiDAR, then multispectral UAV, phenometric data, and structure from motion, and finally RGB‐structure from motion for the mapping of 10 different species, achieving accuracies of 78%, 52%, and 45%, respectively. Using a uniquely cross‐disciplinary approach, Janišová et al. (2024) combined a time series of satellite‐based NDVI going back to 1984 with ground surveys, history, and ethnology for land use change and cultural practices in two villages in the Serbian Carpathian grasslands. By gaining an understanding of the history and culture of the regions, the authors were better informed regarding the historical land management practices, how they have changed, and the influence this has had on current biodiversity levels. This further enhanced their interpretation of the historical NDVI record and allowed the authors to make specific recommendations on land use, such as a partial return to historical land management practices to at least partially restore some of the lost species richness.

## 4 Functional Traits

As a reminder, six above‐ground functional traits (LAI, LDMC, N, P, K and CP) were chosen to represent different functional properties of grasslands, some of which have been surveyed by remote sensed methods extensively in the literature (e.g., 35 research studies on LAI here), and others that have seen many fewer studies (just two studies on K).

### 4.1 Leaf Area Index

The LAI papers total 35, excluding reviews. As only eight deal primarily with hyperspectral sensors, it makes more sense to divide these by the spatial resolution of the sensors: (1) High Resolution for example, UAV and Aircraft (0.01–1.0 m). (2) Medium Resolution for example, Goafen‐2, Landsat, Sentinel‐2 (3–30 m). (3) Low Resolution for example, MODIS and Sentinel‐3 (> 30 m).

#### 4.1.1 High Resolution

UAVs equipped with hyperspectral cameras have been used to measure LAI across 4 studies in Inner Mongolia since 2019. The best results were achieved on a grassland monoculture site with an *R* ^2^ of 0.87 between UAV level canopy measurements and ground sampling through partial least squares regression (PLSR) (Zhao, Sun, Lu, et al. 2021). Using linear regression to relate UAV derived VIs to LAI, Sha et al. (2019) produced an *R* ^2^ of 0.45 between the generalized soil‐adjusted vegetation index and LAI, with more of the errors coming from regions with low LAI values. However, Zhu et al. (2023) used the PROSAIL model to determine the optimum VIs, then used a two‐layer VI matrix to calculate LAI, with an *R* ^2^ of 0.73. Zhu et al. (2024) developed this further over a species rich grassland by using the PROSAIL model and two simple vegetation indices, the optimized soil‐adjusted vegetation index and NDVI, achieving an *R* ^2^ of 0.84.

Two additional studies were carried out focusing on aircraft mounted spectrometers, with contrasting results. In a Tallgrass site in the USA, the National Ecological Observatory Network (NEON) LAI was not significantly related to ground‐based LAI measurements (Pau et al. 2022). With a different approach, Bandopadhyay et al. (2019) found that higher rates of sun‐induced fluorescence at 687 and 760 nm were associated with greater LAI (*R* ^2^ of 0.80 and 0.86 respectively) in their natural test sites in Poland, which included many species‐rich grasslands. The sun‐induced fluorescence measures also correlated well with greenness‐related VIs, such as NDVI.

#### 4.1.2 Medium Resolution

A range of methods has been employed under the medium resolution remote sensing of LAI. Xu et al. (2018) and Qin et al. (2021) compared different VIs to ground‐based LAI measurements, with the perpendicular vegetation index from Landsat and the normalised difference phenology index from Sentinel‐2 performing best, respectively. In two studies using the Copernicus Land Monitoring Service LAI products and Sentinel‐2 in Poland, Dąbrowska‐Zielińska et al. (2024) and (Panek‐Chwastyk et al. 2024) found strong agreements with ground‐based LAI, with *R* ^2^ values of between 0.62 and 0.93.

Machine learning was also the focus of several LAI studies. In South Africa, RF has been used to successfully map LAI with both Landsat and Sentinel‐2, but with slightly stronger results in the dry season versus the wet season (Dube et al. 2019; Masenyama et al. 2023). In more mountainous South African grasslands, Tsele et al. (2023) found that the optimal regression choice, RF or stepwise multiple linear regression, varied depending on the location. Shen et al. (2022) assessed a range of different machine learning approaches (RF, neural networks and support vector regression) on Landsat‐8 data to model LAI, with RFs again tending to produce the most accurate results. Three studies have attempted to use machine learning methods to integrate synthetic aperture radar (SAR) data with multispectral for mapping LAI. Lu and He (2019) found the improvements from including SAR in their RF over the southern Canadian Prairies was marginal. However, Wang et al. (2019) found that SAR data improved LAI estimates in their multiple linear regression model over areas of dense tallgrass vegetation where typical VIs tend to become saturated, a finding supported by a subsequent study of Alpine grasslands in northern Italy (Castelli et al. 2023).

Five studies have used radiative transfer models (RTMs) with medium resolution satellite imagery to aid in mapping LAI. In test farms in southern England, the PROSAIL model was used with Sentinel‐2 for LAI mapping, achieving strong correlations and offering an improvement over LAI calculated from NDVI (Punalekar et al. 2018). In Brazil, the Automated Radiative Transfer Model Operator was used with Sentinel‐2 also. The authors found that the Normalised Area Over Reflectance Curve index produced the strongest results (Cisneros et al. 2020). In Austria, Sentinel‐2 was used with two RTMs for the growing seasons of 2018 and 2019, achieving an *R* ^2^ of 0.87 with direct ground measurements of LAI (Klingler et al. 2020). Similar success was achieved in grassland of northern China using the PROSAIL model again, with an *R* ^2^ of 0.82 between the newly developed Chlorophyll‐Insensitive VI and LAI (Zhang, Jin, et al. 2023). In northeastern Germany, Schwieder et al. (2020) tested the accuracy of two methods for assessing LAI using Sentinel‐2—RF regression and a soil‐leaf‐canopy RTM, with both models demonstrating strong predictive power. Brown et al. (2021) compared a novel Level 2 processor for Sentinel‐2 data, with updated artificial neural networks retrieval methodology. The updated method was close to or better than the old over many vegetation types, but slightly worse over grasslands. Jiang et al. (2024) developed a new bi‐directional reflectance distribution function for the Gaofen‐1 satellite to improve vegetation parameter accuracy with tests over grasslands in northeast China. The new bi‐directional reflectance distribution function produced an *R* ^2^ of 0.58, 0.14 higher than the previous method. In central China, Peng et al. (2024) applied topographic corrections to a large range of LAI models based on Landsat 8 imagery, and compared them to LAI products, such as from MODIS and Global Land Surface Satellite, and ground sampling. Topographic corrections, when combined with RTMs, improve the correlations (*R* ^2^ improvements of 0.18–0.04) and reduce the errors more than machine learning combined with RTMs. They also produced an *R* ^2^ improvement of > 0.2 compared to MODIS and GLASS LAI products. The research is focused on mountainous terrain and so may not be as applicable in flatter grasslands.

#### 4.1.3 Low Resolution

Many low‐resolution global LAI products currently exist and have been used in a large range of studies over recent years. These products include, for example, the MODIS derived MOD15A2 and MOD15A2h, Geoland2 Version 1 (GEOV1) and Global Land Surface Satellite (GLASS), each with different development methods, temporal, and spatial resolutions.

Several recent studies have compared these LAI products with ground measurements and high‐resolution satellite data across different ecosystems and countries. Li, Lu, et al. (2018) compared MOD15A2, GLASS, Global LAI Product of Beijing Normal University, and Global LAI Map of Chinese Academy of Sciences (GLOBMAP) with ground measurements both across the globe and, with special emphasis, over China. Overall, GLASS performed best in both situations, with *R* ^2^ values of 0.70 and 0.94 respectively. Even though grasslands made up 43.1% of the assessment area in China, specific correlations for grasslands are not provided. Another comparison was carried out by Liu et al. (2018) between MOD15A2, GLASS, and the Four‐Scale Geometric Optical Model over a mixture of land cover types in China. The Four‐Scale Geometric Optical Model was found to perform slightly better in grasslands, with an *R* ^2^ of 0.5, 0.09, and 0.22 better than MOD15A2 and GLASS respectively. A specific grassland comparison of GEOV2, GLASS, GLOBMAP, and MOD15A2h was carried out in Inner Mongolia, with GLOBMAP performing best in meadows, GLASS best in typical steppe, and GEOV2 best in desert steppe, but all with *R* ^2^ values below 0.4 (Shen et al. 2023). Yin et al. (2020) demonstrated that the temporal resolution is also important to consider. Comparing MOD15A2, MOD15A2h, GEOV1, and GLASS, the authors found that the MODIS‐based LAI products had lower *R* ^2^ compared to the other datasets, but the shorter temporal window allowed for sudden changes to be detected, while GEOV1 and GLASS had high *R* ^2^ values; they missed grazing‐induced sudden changes due to their broad temporal windows.

Munier et al. (2018) used Kalman filtering to disaggregate global GEOV1 data, allowing them to assign different LAI values to different vegetation types within a single pixel. While producing improvements over most vegetation types, this method reduced the accuracy over grasslands, with the *R* ^2^ dropping from 0.89 to 0.82 compared to the original data. Several attempts have been made to fuse high‐resolution, but temporally sparse, LAI data with low‐resolution global products, but with mixed results. Li, Lu, et al. (2018) used Landsat‐7, ‐8, and Sentinel‐2 to generate 30 m resolution LAI maps in northern China using PROSAIL. These were combined with the MODIS data using a spatial and temporal adaptive reflectance fusion model. The authors note reductions in errors and noise in their new fused datasets, with the *R* ^2^ of 0.62 versus 0.53 for the original MODIS LAI product. Zhou et al. (2020) took a different approach, using a time series of MOD15A2H as a long‐term background signal; ground measurements and Landsat‐7 and ‐8 were fused using a back propagating neural network to create 30 m LAI maps for the study regions in Ukraine and China. A modified ensemble Kalman filter model using both the Landsat and MODIS data allowed for the 30 m LAI to be spread over the space and time of the MODIS data, achieving an *R* ^2^ of 0.88 over grasslands. Across mixed test sites in the USA, another approach to combining field data, MODIS, and Landsat through a deep transfer learning framework failed to produce substantial improvements over grasslands but was successful over croplands and forests (Zhou et al. 2023).

Finally, in northern China, Li et al. (2024) compared the ability of Sentinel‐3 to retrieve vegetation parameters such as LAI, with MODIS and PROBA‐V, using a range of prediction methods. Sentinel‐3 had better accuracy than other platforms, likely due to the red edge bands, but only slightly compared to PROBA‐V (*R* ^2^ of 0.63 each).

### 4.2 Leaf Dry Matter Content

There has been limited success in measuring LDMC over the Tibetan Plateau. Li, Wulf, et al. (2018) attempted to measure plant dry matter content with satellite imagery and use this as a proxy measure for the community weighted mean (CWM) of LDMC. However, the correlation was weak, with an *R* ^2^ of just 0.1. In different parts of the Tibetan Plateau, Zhang et al. (2022) used UAV‐based hyperspectral imagery and ground sampling to map community LDMC through numerous different machine learning models. The generic algorithm integrated with the PLSR performed best for LDMC, although it only explained 30% of the variance. The authors claim that the lack of spectral data at 1450 and 1950 nm, which is linked to water content, reduced the accuracy of their models. In contrast, a different approach to mapping the community weighted mean LDMC was conducted by Polley et al. (2020a) at a restored grasslands site in Texas. Four years of hyperspectral reflectance measurements from the ground and UAVs, as well as ground sampling, were incorporated into a PLSR analysis, with the model explaining 73% of the LDMC of their canopies. Three further studies used the same analysis and LDMC data as Polley et al. (2020a) for different goals, such as looking at what regulates the temporal stability of grassland metacommunities (Polley et al. 2020b), biomass production (Polley et al. 2020) and the influence of community LDMC on plant production (Polley et al. 2022).

### 4.3 Potassium, Phosphorus, and Nitrogen

Of the 24 research (two were reviews) papers that made up the review of Potassium (K), Phosphorus (P) and Nitrogen (N):

- 15 focused on just nitrogen
- 5 focused on both nitrogen and phosphorus
- 2 focused on just phosphorus
- 1 focused on nitrogen and potassium
- 1 focused on nitrogen, phosphorus, and potassium

Due to the overlap in papers measuring these plant nutrients, N, P, and K have been grouped together, and then split in multispectral remote sensing methods and hyperspectral methods.

#### 4.3.1 Multispectral

Across lowland experimental sites, strong N prediction accuracies were achieved using both satellite (Smith et al. 2023) and UAVs (Grüner et al. 2021; Oliveira et al. 2022) in combination with machine learning, especially RFs. Using the simpler Three Band Index from Sentinel‐2, Smith et al. (2023) achieved poorer results, with an accuracy of just 38%. Managed grassland sites also saw strong correlations with both N and P with satellite data (Morais et al. 2023) and for N with UAV data (Lussem et al. 2022) both using RFs. Only moderate predictive strength for N was found using Landsat and Sentinel‐2 data with a RTM in dairy farms in New Zealand (Dehghan‐Shoar et al. 2023), and Schucknecht et al. (2022) achieved an *r* ^2^ of 0.47 for N in mixed management sites in southern Germany using UAV data and RFs.

Much of the research using multispectral surveys of natural grasslands has occurred in alpine regions, particularly in China. While Tang et al. (2021) failed to map N content accurately using a UAV mounted digital camera, Gao et al. (2020) achieved moderate success in mapping the N/P ratio over the Tibetan Plateau (*R* ^2^ of 0.49 in July, and 0.59 in November) using Sentinel‐2 and RFs. Very strong results were obtained with satellite imagery in measuring P (Shi et al. 2024), N, P, and K (Zhang, Liang, et al. 2023) and N and P (Pang et al. 2022) with *r* ^2^ values of 0.74–0.85, using methods ranging from graph theory to create pseudo‐hyperspectral images from Sentinel‐2 to RFs and PLSR, respectively.

#### 4.3.2 Hyperspectral

One study attempted to incorporate hyperspectral satellite observations for measuring P in the West African Savanna, but failed to achieve any significant correlations (Ferner et al. 2021), with the authors suggesting that low levels of P meant that other features dominated the absorption spectra, and no other features capable of being remotely sensed correlated with P in the study area, rendering their attempts unsuccessful. The remaining studies utilized aircraft and UAV‐mounted hyperspectral instruments but with mixed results—six over experimental sites and three over natural grasslands. Of the experimental sites, one measured both N and P at an Inner Mongolian test site, achieving *R* ^2^ values of 0.87 and 0.54, respectively (Zhao, Sun, Lu, et al. 2021)—here the authors claim the lower accuracy for P was due to a lack of spectral bands that P primarily absorbs. The remaining five experimental sites measured just N and achieved *R* ^2^ values from 0.57 to 0.87, using methods from basic regression (Polley et al. 2023) to PLSR (Cavender‐Bares et al. 2022; Franceschini et al. 2022; Wang et al. 2019; Zhao, Sun, Lu, et al. 2021) and machine learning (Oliveira et al. 2020). For the remaining three studies that focused on natural grasslands, Pau et al. (2022) found the N concentration product of the NEON surveys compared poorly to ground sampling, which the authors claim was due in part to the high levels of biodiversity at the site. Similarly, while Gholizadeh, Friedman, et al. (2022) achieved *R* ^2^ values of between 0.51 and 0.62 for K and 0.42 and 0.50 for N, they also claimed that results were worst in species‐rich grasses. Finally, Zhang et al. (2022) achieved similar results using UAV hyperspectral data and a GA‐PLSR model over natural grassland on the Tibetan Plateau, with an *R* ^2^ of 0.50 and 0.54 for community‐level N and P.

### 4.4 Crude Protein

CP has been successfully mapped across a both local and regional scales using satellites and, increasingly, UAVs. Low‐resolution MODIS imagery has been used to predict CP with high levels of accuracy in Tibet with RFs (Han et al. 2022), while medium‐resolution Sentinel‐2 accurately mapped CP in both Germany and Mongolia, using RFs (Raab et al. 2020; Zhao et al. 2023) and high‐resolution PlanetScope imagery was used with RFs to successfully map CP across tropical grasslands in Colombia (Zwick et al. 2024).

Aside from the study by Hart et al. (2020), where the authors failed to generate a strong correlation between UAV multispectral data and CP in commercial grasslands (due, as the authors claim, to their model being tuned to a different type of grassland), UAVs have shown great accuracy in mapping CP at local scales. Gao et al. (2019) used simple band indices to accurately map CP in natural grasslands in China, while in a Colombian grazed grassland, Giraldo et al. (2023) also used simple band indices to map CP, improving further on the accuracy through generalized additive models. Machine learning has also been employed to predict CP using UAV‐derived hyperspectral surveys with high accuracy (*R* ^2^ > 0.70) from experimental sites in southern Norway (Geipel et al. 2021) to tropical rangelands in Northern Australia (Barnetson et al. 2020) and mixed management grasslands in central Germany (Wijesingha et al. 2020). Finally, in an experimental test site in Ireland, Askari et al. (2019) compared measurements from a handheld hyperspectral device, UAV multispectral, and Sentinel‐2 images. While the handheld multispectral device produced the best results, the UAV also showed very strong predictive value and with better accuracy than the satellite measurements.

## 5 Discussion and Recommendations

### 5.1 Biodiversity

Recent years have seen significant advancements in access to free, moderate to high‐resolution multispectral satellite imagery, alongside the rapid development of UAV technology allowing both multispectral and hyperspectral data to be captured across a huge range of spatial resolutions and scales. However, evidence from the papers covered in this review shows that there are still substantial uncertainties regarding how best to connect the spectral diversity to species diversity, with significant variability in the correlations achieved. This uncertainty appears to exist regardless of whether the measurements occur with hyperspectral or multispectral instruments, regardless of the spatial scale, the spatial resolution, location, or type of spectral variation metric tested. This is supported by a 2023 metanalysis that found an average correlation of just 0.36 between spectral variation and species diversity in grasslands, with significant variability occurring both within and between studies (Thornley et al. 2023). Another systematic review of related papers between 2000 and 2022 suggested that more work needs to be done to identify factors that influence the SVH (Lyu et al. 2024), while another study highlights that many factors can exert a greater influence on SD than just species diversity, limiting the situations where the SVH can be successfully applied (Fassnacht et al. 2022). Machine learning algorithms have emerged as new and effective tools for mapping grassland biodiversity but, like the SVH, this method needs to begin better accounting for sources of error and uncertainty. As such, we present some recommendations regarding the remote sensing of biodiversity:

- Bare soil can weaken the spectral signal and thereby reduce the correlations between SD metrics and species diversity. It is necessary to filter out bare soil pixels from remotely sensed imagery where possible (Kamaraj et al. 2024; Rossi and Gholizadeh 2023; Xu et al. 2022).
- In areas of low biomass, dead biomass can also alter the reflected spectral values, influencing the SD metrics generated. Where possible, the proportion of live and dead biomass should be measured and factored into the analysis (Rossi et al. 2022).
- The vegetation phenological stage influences the interaction of plants with light and so exerts a significant influence on their spectral signatures. Generating a time‐series of spectral diversity can help to account for these variations (Hall and Lara 2022; Perrone et al. 2024; Rossi et al. 2021).
- The vertical complexity of the vegetation structure can reverse the relationship between SD and species diversity (Conti et al. 2021), while combining 3D vegetation data with SD has been shown to improve correlations with species diversity (Hall and Laura., 2022). It is beneficial to incorporate 3D vegetation data, from structure from motion or LiDAR, into the study workflow.
- Several studies suggest spatial resolutions of 1 mm (Wang et al. 2018) to 1 m (Gholizadeh et al. 2020) tend to work best, but this does seem site dependent. Therefore, when using UAVs, a range of survey heights should be tested to ensure the best results.
- Ground sampling design and plot sizes should be adjusted based on the pixel size and area of the remote sensing survey. This can ensure the best match is achieved between the species count on the ground and the spectral diversity metrics extracted from the remote sensing survey (Gholizadeh, Dixon, et al. 2022; Rossi and Gholizadeh 2023).
- Machine learning can be an effective tool for measuring species diversity, especially random forests regression. However, more work needs to be done to uncover the sources of error and variability present in the published literature.
- For a long‐term analysis of changing grassland biodiversity, understanding the local cultural and historical practices that influence land management styles can provide important context in understanding current biodiversity and interpreting long‐term datasets (Janišová et al. 2024).

Finally, integrated long‐term surveys in a range of different grassland environments, linking ground data collection, low‐elevation aerial observations, high‐elevation aerial observations, and satellite observations, should be combined with SD analysis and machine learning. This can best account for temporal and spatial scale discrepancies, thereby allowing for the analysis to more effectively identify the most suitable spectral diversity metrics and regression/machine learning tools to model biodiversity.

### 5.2 Functional Traits

#### 5.2.1 Leaf Area Index

Linking high‐resolution, hyperspectral data from aircraft and UAVs to LAI is a growing area of research. The data coming from these platforms appear to be capable of modeling LAI with high degrees of accuracy, with three of the five studies reviewed having *R* ^2^ values of 0.73 or higher. These studies have achieved success using analysis as basic as linear regression up to RTMs and machine learning, and over both experimental and natural sites. The NEON LAI product tested by Pau et al. (2022) is again the worst performing. However, all the successful studies were performed at just local scales with extensive ground validation, versus the NEON aerial observation platform product, which is based on large scale aerial surveys with little to no ground validation of the product.

A wide variety of approaches have been used with medium‐resolution, multispectral satellite data. The most successful appears to be those focused on radiative transfer models (*n* = 5), with an average *R* ^2^ of 0.75 and a range from 0.57 to 0.87. Studies primarily relying on machine learning models (*n* = 8) have generally proven effective too, with an average *R* ^2^ of 0.67, ranging from 0.46 to 0.87. However, those high and low values come from two studies, Masenyama et al. (2023) and Tsele et al. (2023), and both using Sentinel‐2 and both based in mountainous regions of South Africa. This hints at the possibility of additional uncertainties being added to surveys in mountainous terrain. Indeed, the work of Peng et al. (2024) showed that applying a topographic correction to Landsat‐8 improved the correlations and reduced the errors from both RTM‐derived and machine learning‐derived LAI data. Furthermore, the addition of SAR data was found to improve prediction accuracy in regions where vegetation indices approach saturation (Castelli et al. 2023; Wang et al. 2019).

Despite three studies performing intercomparisons between global LAI products since 2018, no product performs consistently better than any other, and *R* ^2^ values vary significantly from one comparison to the next (Li, Lu, et al. 2018; Liu et al. 2018; Shen et al. 2023). Even products with greater spatial resolution often require a broader temporal window for complete daily coverage, meaning that transient or sharp changes in LAI can be missed (Yin et al. 2020). Attempts by Munier et al. (2018) to extract sub‐pixel LAI values failed to provide an accuracy improvement over grasslands, despite working for other vegetation types. Recent efforts to fuse finer resolution data from Landsat and Sentinel‐2 with global LAI products such as those from MODIS have produced mixed results thus far over grasslands (Li, Huang, et al. 2018; Zhou et al. 2020, 2023). As such, further development of these fusion models will be necessary before a reliable global LAI model with both high spatial and temporal resolution can be distributed.

- LAI mapping from high‐resolution hyperspectral surveys can be successful using a variety of regression and modeling approaches, but parameters need to be tuned to specific regions to ensure accuracy. Research integrating hyperspectral satellite measurements should aid in this task.
- New research demonstrated that sun‐induced fluorescence at 687 and 760 nm has a strong association with LAI, potentially opening the door to a new form of high‐resolution LAI mapping.
- With moderate resolution multispectral data, both RTMs, especially PROSAIL, and machine learning methods, particularly random forests, have produced consistent and robust estimations of LAI.
- Moderate resolution LAI estimates may also benefit from topographic corrections in more rugged terrain and the addition of SAR data where vegetation indices become saturated.
- Comparisons of global LAI products have failed to identify a single best option, and attempts to fuse high‐ and low‐resolution data are still in development and lack consistency. It is therefore necessary to test a range of products to find the one most suited to the study area in question and with the necessary spatial and temporal resolution.

#### 5.2.2 Leaf Dry Matter Content

Little progress has been made in the remote sensing of grassland LDMC, although the work of Polley et al. (2020a) demonstrated that a significant correlation with LDMC could be established with UAV‐based hyperspectral imagery using PLSR. This was backed up by Zhang et al. (2022) using a similar method but only achieved a weaker, though still significant, relationship with LDMC. PLSR and hyperspectral remote sensing appear to show promise in mapping LDMC, but this approach is in its early stages and much more work is required.

- Initial studies by Polley et al. (2020a) and Zhang et al. (2022) show that UAV‐based hyperspectral UAV data and PLSR have the potential to predict LDM content, but more work needs to be done to exploit this method and determine the optimal spectral bands required to model LDMC.

#### 5.2.3 Potassium, Phosphorous, and Nitrogen

The two studies dealing with Potassium, Zhang, Liang, et al. (2023) and Gholizadeh, Friedman, et al. (2022), used different observation platforms and analysis methods but both with strong results and in natural settings. While there has not been enough research on remote sensing of K in grasslands, the two results shown suggest that it is feasible, whether across a broad landscape using multispectral satellite data or over a small region using aircraft‐mounted hyperspectral data. However, more work needs to be done to assess the range, consistency, and applicability of these measurement tools, and ensure that these methods are robust.

For the seven studies that provided estimates of P, the four that used multispectral satellite surveys achieved an average *R* ^2^ value of 0.78, while the three that used hyperspectral imagery (two UAV based, one satellite‐based) averaged just 0.4. Two of the hyperspectral studies (Zhang et al. 2022; Zhao, Sun, Lu, et al. 2021) suggested that they lacked the appropriate bands in their spectrometers to accurately measure P. While it is difficult to infer anything significant given the sparse number of studies, these results stand in contrast to the review by Van Cleemput et al. (2018), which found an average *R* ^2^ of 0.75 for the hyperspectral remote sensing of Phosphorous in grass‐ and shrublands. This suggests there are still substantial uncertainties that need to be addressed regarding the remote sensing of P, especially using hyperspectral imagery. With the greater availability and affordability of UAV‐based multi‐ and hyperspectral instruments, avenues are now available to investigate the potential spatial and temporal sources of uncertainty within these measurements.

The studies measuring Nitrogen produce more consistent results than P, with an average *R* ^2^ 0.63 and 0.62 for multispectral and hyperspectral measurements, respectively. This is more in line with Van Cleemput et al. (2018), who found an average *R* ^2^ of 0.74, but that included proximal measurements that are likely to be more accurate. No significant difference arises from the platform used (UAV, Aircraft or Satellite), the regression or modeling approach, nor the study location. Some studies did fail to establish a significant relationship, such as Tang et al. (2021) using a standard camera on a UAV, or Pau et al. (2022) when assessing the NEON aircraft‐based hyperspectral products. Furthermore, no studies made use of hyperspectral satellite data, with all hyperspectral nitrogen remote sensing being based on either UAV or aircraft surveys.

- Additional research needs to be done to build on the initial success of studies mapping grassland potassium and to assess their range and limits of applicability.
- Recent studies covering the remote sensing of phosphorus show a greater level of variability in the hyperspectral measurements than multispectral. Some studies suggest that regions with high levels of biodiversity produce weaker results, and other studies appeared to lack the appropriate spectral bands to effectively map P. This area requires further study to identify and mitigate the sources of uncertainty.
- Studies measuring grassland nitrogen content appear more consistent and robust than K and P. However, a few still fail to establish strong correlations. This suggests extracting suitable values may still require fine‐tuning based on local or regional vegetation characteristics.
- K, P and N values in grasslands would benefit from greater use of satellite‐based hyperspectral data, especially when used in conjunction with aerial surveys for multi‐spatial and ‐temporal scale analysis.

#### 5.2.4 Crude Protein

Of the 11 studies presented on the remote sensing of CP, eight used primarily multispectral data (from both UAV and satellites) and three used hyperspectral UAV data. Six of those 11 in total incorporated machine learning methods. However, barring one exception (Hart et al. 2020) all studies found strong and significant correlations with CP, often using just simple band ratios and/or VIs with a mix of different regression algorithms. Given the ease of use, low costs, and effectiveness of consumer‐grade multispectral UAVs and free multispectral satellite imagery, the tools and data for mapping of grassland CP content from centimetre to decametre scales are becoming increasingly accessible to a growing range of researchers and land managers.

- Crude protein can be effectively measured from both multi‐ and hyper‐spectral data, at large and small spatial scales, and with a range of simple and complex modeling methods. The studies presented here have exclusively focused on local or regional sites. Therefore, the feasibility of scaling these surveys to national or global levels should be assessed in the near future.

## 6 Conclusions

This review has examined the remote sensing of grassland biodiversity and six functional traits with a focus on recent technological and methodological developments. Advances in UAV technology have accelerated the increase in grassland surveys featuring very high spatial resolutions, with 3D components and increasingly employing multispectral and hyperspectral sensors. While much progress has been made in identifying confounding factors and sources of variability regarding the application of the SVH to biodiversity mapping, significant uncertainties still exist that place substantial limitations on this methodology. At the same time, machine learning methods are becoming prevalent within the research community, requiring a strong understanding of the many parameters needed to construct a useful statistical or predictive model. While these developments open the door to new approaches and new discoveries, they also present new sources of error and uncertainty. This requires a more structured and systematic approach to investigating, documenting, and addressing these issues. Utilizing UAV surveys as a bridge between point‐based groundwork and satellite remote sensing, helping to integrate measurements across spatial and temporal scales, is one step in this process that can be implemented in many locations across the planet. This could be further enhanced by making better use of currently existing and future hyperspectral satellite platforms, such as EnMAP, PRISMA, and the Firefly constellation. Finally, to develop more generalizable remote sensing models for biodiversity and grassland functional traits (e.g., Serbin et al. 2019), we stress the importance of an open, global database, similar to TRY (Kattge et al. 2011) of traits, species, and related spectra from multi‐ and hyperspectral devices. This could provide a structured foundational database to the remote sensing of both grassland biodiversity and functional traits—allowing for greater systematic error and uncertainty analysis, reducing redundancy, and accelerating the development of more robust, scalable, and generalizable remote sensing models.

## Author Contributions

**Samuel Hayes:** conceptualization (lead), data curation (lead), formal analysis (lead), investigation (lead), methodology (lead), visualization (lead), writing – original draft (lead), writing – review and editing (lead). **Fiona Cawkwell:** funding acquisition (supporting), writing – original draft (supporting), writing – review and editing (supporting). **Karen L. Bacon:** funding acquisition (supporting), writing – original draft (supporting), writing – review and editing (supporting). **Astrid Wingler:** funding acquisition (lead), writing – original draft (supporting), writing – review and editing (supporting).

## Conflicts of Interest

The authors declare no conflicts of interest.

## Data Availability

The method used for gathering the papers reviewed in this article can be found in Section 2 and Figure 2. These, and additional papers, are all included in the reference list.
